# Expression Profiling of Major Histocompatibility and Natural Killer Complex Genes Reveals Candidates for Controlling Risk of Graft versus Host Disease

**DOI:** 10.1371/journal.pone.0016582

**Published:** 2011-01-28

**Authors:** Peter Novota, Severin Zinöcker, Jean Norden, Xiao Nong Wang, Lisbet Sviland, Lennart Opitz, Gabriela Salinas-Riester, Bent Rolstad, Anne M. Dickinson, Lutz Walter, Ralf Dressel

**Affiliations:** 1 Department of Cellular and Molecular Immunology, University of Göttingen, Göttingen, Germany; 2 Department of Anatomy, Institute of Basic Medical Sciences, University of Oslo, Oslo, Norway; 3 Haematological Sciences, Institute of Cellular Medicine, Newcastle University, Newcastle-upon-Tyne, United Kingdom; 4 Department of Pathology, Haukeland Sykehus, Section of Pathology, Gades Institute, University of Bergen, Bergen, Norway; 5 Transcriptome Analysis Laboratory, University of Göttingen, Göttingen, Germany; 6 Department of Primate Genetics, German Primate Center, Göttingen, Germany; Université de Toulouse, France

## Abstract

**Background:**

The major histocompatibility complex (MHC) is the most important genomic region that contributes to the risk of graft versus host disease (GVHD) after haematopoietic stem cell transplantation. Matching of MHC class I and II genes is essential for the success of transplantation. However, the MHC contains additional genes that also contribute to the risk of developing acute GVHD. It is difficult to identify these genes by genetic association studies alone due to linkage disequilibrium in this region. Therefore, we aimed to identify MHC genes and other genes involved in the pathophysiology of GVHD by mRNA expression profiling.

**Methodology/Principal Findings:**

To reduce the complexity of the task, we used genetically well-defined rat inbred strains and a rat skin explant assay, an *in-vitro*-model of the graft versus host reaction (GVHR), to analyze the expression of MHC, natural killer complex (NKC), and other genes in cutaneous GVHR. We observed a statistically significant and strong up or down regulation of 11 MHC, 6 NKC, and 168 genes encoded in other genomic regions, i.e. 4.9%, 14.0%, and 2.6% of the tested genes respectively. The regulation of 7 selected MHC and 3 NKC genes was confirmed by quantitative real-time PCR and in independent skin explant assays. In addition, similar regulations of most of the selected genes were observed in GVHD-affected skin lesions of transplanted rats and in human skin explant assays.

**Conclusions/Significance:**

We identified rat and human MHC and NKC genes that are regulated during GVHR in skin explant assays and could therefore serve as biomarkers for GVHD. Several of the respective human genes, including *HLA-DMB*, *C2*, *AIF1*, *SPR1*, *UBD*, and *OLR1*, are polymorphic. These candidates may therefore contribute to the genetic risk of GVHD in patients.

## Introduction

Haematopoietic stem cell transplantation (HSCT) is currently the only potentially curative treatment for many malignant and non-malignant haematological diseases. However, the overall survival rate after transplantation is still only 40% to 60% due to severe posttransplant complications [1], which include graft versus host disease (GVHD), relapse, and infection. Human leukocyte antigen (HLA) matching is essential to reduce the risk of graft rejection and GVHD [2]. However, non-HLA genes also impact on transplant outcome [3] and acute GVHD can be fatal even in patients receiving transplants from HLA-identical matched sibling donors (MSD). The cumulative incidence of grade II to IV GVHD was 35% in a recent study evaluating 1960 MSD transplants [4]. MSDs are currently available for about one third of the patients and, therefore, alternative donors are needed. HLA-matched unrelated donors (MUD) are more readily accepted than cord blood or mismatched related donors.

The level of HLA matching used for selection of MUDs has changed over time and usually includes now *HLA-A*, *B*, *C*, and *DRB1* loci (8/8 match). In some studies matching has been extended to the *HLA-DQB1* locus (10/10 match). A large recent study has compared MSD and 8/8 matched MUD transplants in a homogenous cohort of patients with chronic myeloid leukemia and found a 2.44 times higher risk of grade II to IV acute GVHD in 8/8 matched MUD compared to MSD transplants [5]. In another study, the incidence of grade II to IV acute GVHD was still higher in 10/10 matched MUD compared to MSD transplants [6]. The higher risk of GVHD after MUD compared to MSD transplants could be due to a higher degree of similarity in non-HLA genes for siblings, who share 50% of their genome with the respective recipient. However, also the HLA region itself could contribute to this difference since it harbors, in addition to the classical HLA class I and II genes, more than 200 other genes [7], many with immunological functions. In accordance with this hypothesis, mismatching of microsatellite markers in HLA class I, class II, and class III regions was associated with an increased risk of death in 10/10 matched MUD transplants [8]. The HLA complex, as is the whole human genome, is organized into segments of closely linked genetic variants that are inherited as haplotypes on the same DNA strand [9]. HLA haplotypes can be defined by HLA class I and II alleles and they are in strong linkage disequilibrium with defined genetic variants of non-class I/non-class II genes within the haplotype blocks within this region [10], [11]. However, a given HLA allele can occur in the context of different HLA haplotypes in MUDs. Interestingly, HLA haplotype mismatching in 10/10 matched MUD transplants was associated with an increased risk of severe acute GVHD [12]. This finding demonstrates that the HLA complex encodes in addition to *HLA-A*, *B*, *C*, *DRB1*, and *DQB1* further unidentified genes that contribute significantly to the risk of developing acute GVHD. In case of disparity between donor and recipient alleles these genes may function as minor histocompatibility antigens. Alternatively, specific allelic variants may also increase the risk of GVHD, e.g., *TNFA*, a gene located within the class III region of the MHC encoding the pro-inflammatory cytokine tumor necrosis factor alpha (TNF-α.) Several *TNFA* polymorphisms have been associated with an increased risk of GVHD and some of them are associated with increased TNF-α levels [13]. The strong linkage disequilibrium within the HLA complex makes it very difficult to identify further non-class I/non-class II HLA genes involved in the pathophysiology of GVHD by genetic association studies alone.

HLA gene expression profiling may be an alternative strategy to identify HLA genes that are involved in the pathophysiology of GVHD. We assumed that at least some non-class I/non-class II HLA genes that contribute to the risk of GVHD change their expression levels during disease progression. However, the genetic variation between clinical samples complicates the situation because allelic variation of gene expression could interfere with expression change in the pathophysiological process. Therefore, we decided to analyze a rat model of GVHD making use of genetically well-defined inbred strains. Importantly, the non-class I/non-class II genes of human (HLA) and rat (RT1) MHCs are highly conserved [14], [15], [16]. However, the size and organization of MHC class I encoding regions are considerably variable and the rat possesses a significant number of MHC class Ib genes for which no human homologues exist [15]. At least some of these genes have already been proven to encode ligands for inhibitory or activating natural killer (NK) receptors [17], [18]. In the rat, in contrast to human, NK receptors of the Ly49 killer cell lectin-like receptor type predominate over killer cell Ig-like receptor genes [19]. Therefore, we also included the natural killer complex (NKC) in the expression profiling which harbors the Ly49 genes and additional natural cytotoxicity receptor genes.

To reduce the complexity of the experimental approach, we used an *in-vitro-*model of the graft versus host reaction (GVHR) – the skin explant assay. This assay has been shown to be a sensitive predictor of GVHD in patients [20]. It was also used to study the pathophysiology of GVHR [21]. Recently, we developed a rat skin explant assay [22]. This standardized *in-vitro-*model allows the study of gene expression during GVHR in a setting that is not influenced by undefined genetic differences between tissue samples which is unavoidable in human studies. In the present study we used this model to analyze the MHC and NKC gene expression profiles of GVHR.

## Results

### GVHR in rat skin explant assays

**Figure 1:**
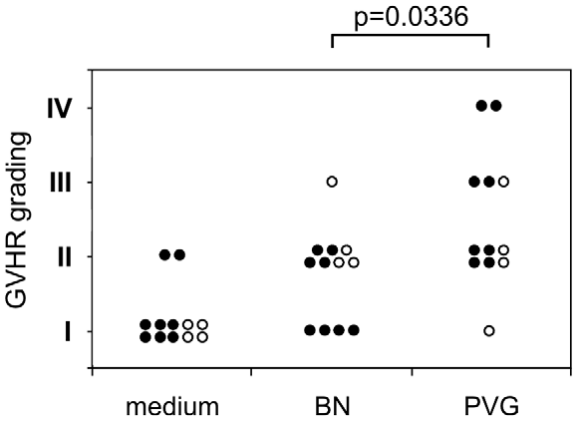
Induction of a GVHR in BN rat skin explants exposed to PVG lymphocytes. A summary of the histological GVHR grading of BN skin samples cultured in medium alone, together with syngeneic BN lymphocytes, and together with pre-stimulated allogeneic PVG lymphocytes (n = 12 in each group) is given. The samples represented by closed circles were used for both gene expression profiling and qRT-PCR experiments, whereas the other samples were only used for gene expression profiling. The pair-wise comparison (U test) indicated a significant difference between skin explant cultures with BN and PVG lymphocytes.

To obtain skin explant samples for an expression profiling experiment, we used BN rats (*RT1^n^*) as recipients and PVG rats (*RT1^c^*) as donors. This combination is mismatched for minor and major histocompatibility antigens, which gives rise to GVHR grades I to IV [22]. PVG splenocytes were stimulated for 7 days in a mixed lymphocyte reaction (MLR) with irradiated BN splenocytes. Syngeneic co-cultures (BN plus irradiated BN splenocytes) were performed as control experiments. The stimulation index indicated a specific proliferation of PVG lymphocytes in response to irradiated BN lymphocytes in contrast to syngeneic cultures of BN lymphocytes (p<0.0001, U test; n = 12 responder animals per strain, data not shown). After 7 days the PVG and BN lymphocytes were harvested, added to fresh BN skin samples from 12 individual animals and cultured for 3 further days. For further controls, additional BN skin samples from the same animals were cultured in medium only. On day 3 the skin samples were harvested and snap frozen for RNA preparation. Parallel samples were fixed and assayed for histological evidence of GVHR (**Fig. 1**). Co-culture of BN skin explants with pre-stimulated allogeneic PVG lymphocytes resulted in higher grade GVHR than co-culture with BN lymphocytes (p = 0.0336; U test). As in a previous experimental series [22], the syngeneic lymphocyte co-culture more frequently resulted in GVHR-like pathology of grade II or higher than culture of the skin explants in medium only.

### Expression profiling of GVHR in rat skin explants

**Figure 2:**
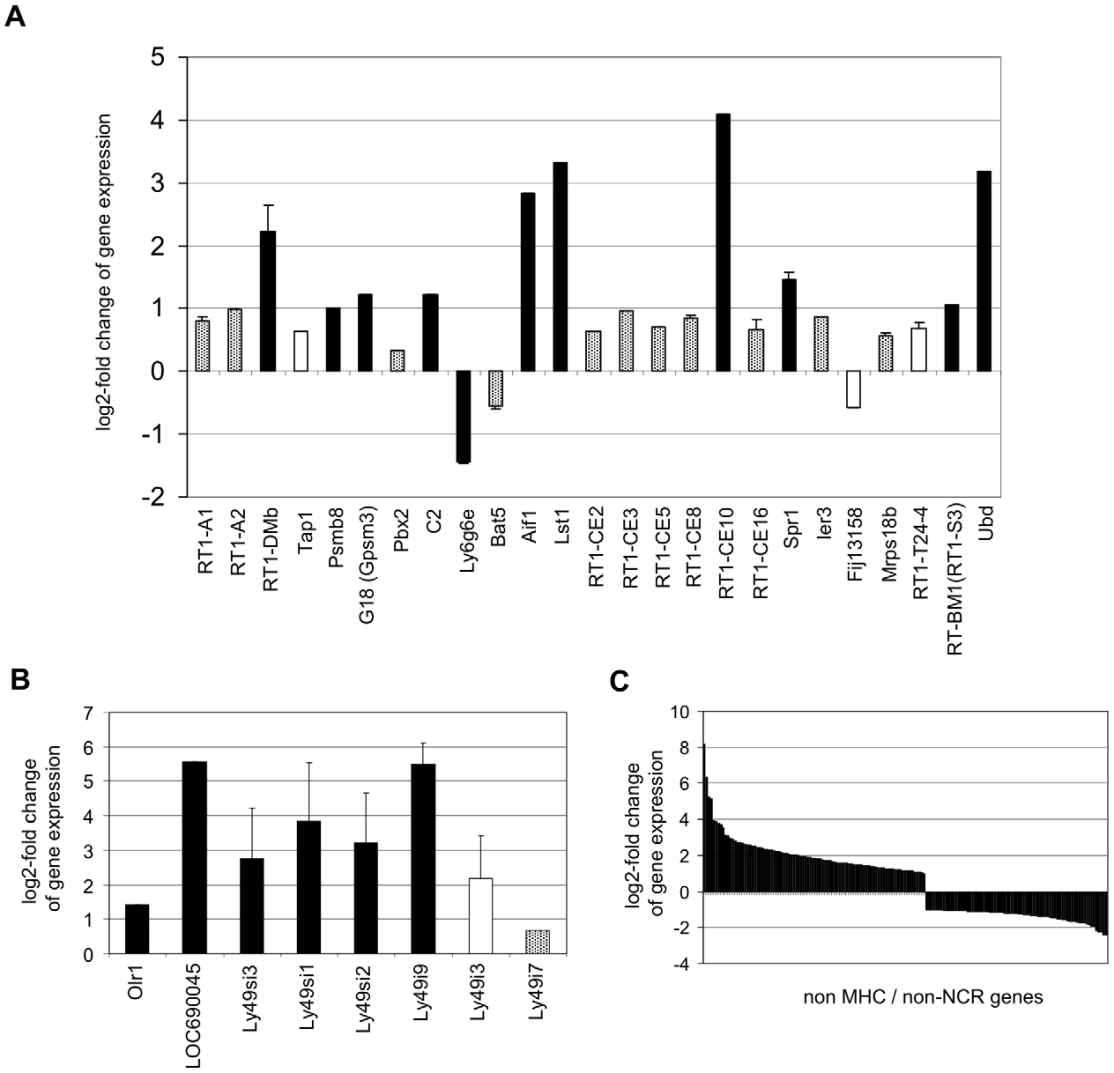
Expression profiling of BN skin explant samples exposed to allogeneic (PVG) lymphocytes in comparison to those exposed to syngeneic (BN) lymphocytes. (*A*) The log2-fold changes in gene expression of significantly regulated MHC genes (p<0.05) are shown. (*B*) The log2-fold changes in gene expression of significantly regulated NKC genes (p<0.05) are shown. (*C*) The log2-fold changes in gene expression of 168 significantly (p<0.05) and strongly (log2-fold change ≥1 or ≤−1) regulated non-MHC and non-NKC genes indicate the range of observed alterations in gene expression levels among the 6342 tested genes. In panels *A* and *B*, black bars indicate a strong change (log2-fold change ≥1 or ≤−1), dotted bars alterations below this amplitude, and white bars expression changes that were not detected at a significant level with all, but at least with 50% of the probes present on the array for that gene. When more than one probe indicated a significant change of gene expression the means and standard deviations of the log2-fold changes are shown (see Tables S1, S2, and S3 for further details).

RNA was prepared from the 24 BN skin explants exposed either to syngeneic (BN; n = 12) or to allogeneic (PVG, n = 12) lymphocytes and used for MHC gene expression profiling. We designed a custom microarray which contained specific probes for the 224 MHC genes. For this purpose the annotated sequence of the MHC of the BN strain was used [16]. For 88 of these genes, i.e. 39.3%, we had to design custom probes. A list of the MHC genes in the chromosomal order with all results obtained in the expression profiling experiment is given in the **Table S1a**. For 42 of the 224 MHC genes, a probe on the array indicated a significant regulation (p<0.05) in the allogeneic skin explant assays (n = 12) compared to the syngeneic controls (n = 12) (**Table S1b**). Eleven of these MHC genes showed on average at least a 2-fold up-regulation (log2-fold change ≥1) or 50% reduction (log2-fold change ≤−1) of mRNA levels (**Fig. 2** ***A***, **Table S1c**). This amplitude of change is conventionally considered to be biologically relevant. Of these genes one was down-regulated (*Ly6g6e*) while 10 were up-regulated (**Fig. 2** ***A***). Fourteen further MHC genes were regulated significantly (p<0.05) but with smaller amplitude (**Table S1c**). The regulation of 17 MHC genes appeared to be more doubtful because less than 50% of the probes for that gene indicated a significant regulation. Thus, we considered 25 MHC genes to be significantly regulated in the expression profiling experiment (**Fig. 2** ***A***). These included the classical class Ia genes *RT1-A1* and *RT1-A2*, 8 non-classical class Ib genes (*RT1-CE2*, *RT1-CE3*, *RT1-CE5*, *RT1-CE8*, *RT1-CE10*, *RT1-CE16*, *RT1-T24-4*, *RT-BM1*) and 3 genes involved in antigen presentation (*RT1-DMb*, *Tap1*, *Psmb8*).

Furthermore, 43 genes of the NKC region, as a second important immune gene cluster, were represented on the array including all *Ly49* genes in this region (**Table S2a**). For 8 of the 43 NKC genes represented on the array, a probe indicated a significant regulation (p<0.05) in the allogeneic skin explant assays compared to the syngeneic controls (**Table S2b, S2c**). In addition to the *Olr1* gene, 6 *Ly49* genes appeared to be up-regulated in the allogeneic skin explant assays (**Fig. 2** ***B***). Not all probes for the *Ly49i3* gene indicated a significant up-regulation. However, all significant results for this gene indicated a strong regulation (log2-fold change >2). A statistically significant (p<0.05) but only moderate up-regulation (log2-fold change <1) was detected for the *Ly49i7* gene.

**Figure 3:**
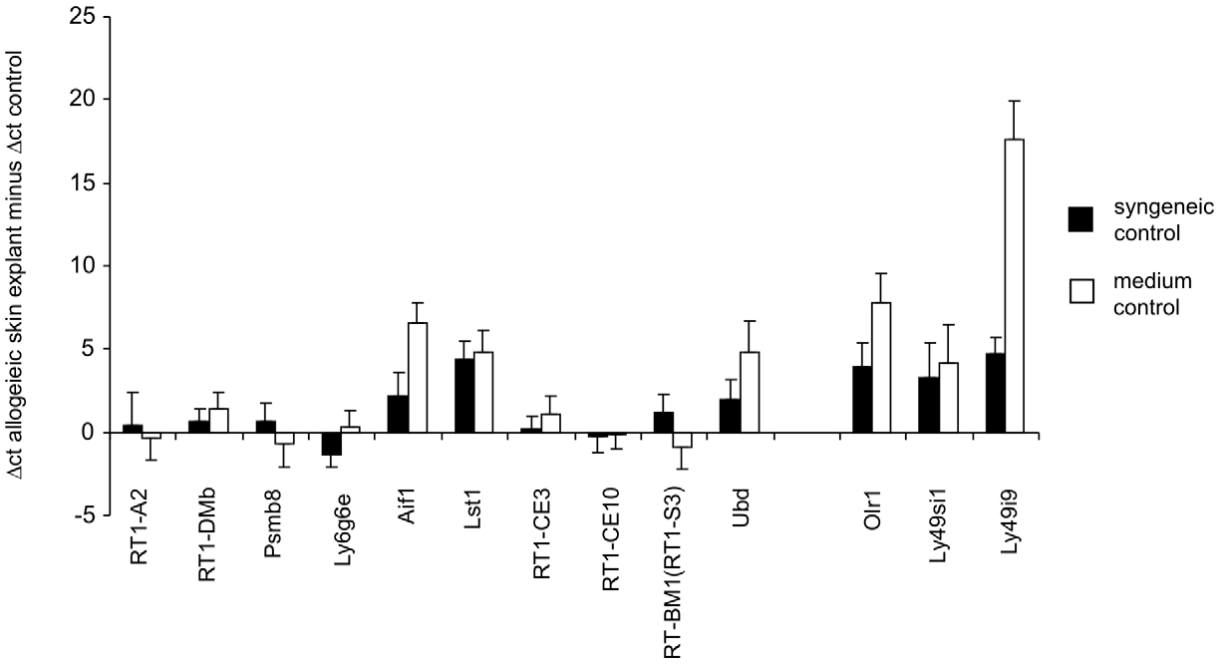
Verification of the regulation in gene expression observed in the microarray experiment by qRT-PCR. A subgroup of 8 samples used for the microarray experiment (see **Fig. 1**) was analyzed by qRT-PCR for the expression of 10 MHC and 3 NKC genes. The ΔΔct value was calculated, i.e. the Δct (*Gapdh* – gene of interest) of the allogeneic skin explant samples minus Δct (*Gapdh* – gene of interest) of the corresponding control sample. The control sample was either a parallel skin explant exposed to syngeneic lymphocytes as in the microarray experiment (syngeneic control, black bars) or a parallel skin explant sample cultured in medium only (medium control, white bars). The means of the ΔΔct values plus standard errors of the mean (SEM) are shown. A positive value indicates an up-regulation of gene expression in the allogeneic samples.

Probes for 6342 additional genes from all chromosomes were included mainly to allow for data normalization. For 168 of the non-MHC/non-NKC genes, a probe on the array indicated a significant (p<0.05) and strong (log2-fold change ≥1 or ≤−1) regulation in the allogeneic skin explant assays compared to the syngeneic controls (**Fig. 3** ***C***, **Table S3**). The 20 genes showing the strongest change in expression levels are shown in **Table 1**. All 20 genes were up-regulated and they included several genes with functions clearly associated with the immune response such as genes encoding chemokines (*Ccl9*, *Ccl6*), Fc receptors (*Fcgr3a*, *Fcgr2b*), the proteases cathepsin S (*Ctss*) and granzyme C (*Gzmc*), and the inflammatory triggering receptor on myeloid cells 2 (*Trem2*).

**Table 1:** The 20 most strongly regulated non-MHC/non-NKC genes in allogeneic skin explants compared to syngeneic controls as revealed by the microarray experiment.

gene	log2-fold change	adjusted p-value	gene description
LOC685020	8.18	0.0100	paired immunoglobin-like type 2 receptor alpha
Ptpns1l3	6.36	0.0100	protein tyrosine phosphatase, non-receptor type substrate 1-like 3
Fcgr3a	5.24	0.0100	Fc fragment of IgG, low affinity IIIa, receptor
Nat8	5.14	0.0100	Rattus norvegicus endogenous retrovirus mRNA, partial sequence [AY212271]
Ccl9	4.16	0.0100	chemokine (C-C motif) ligand 9
XM_226926	3.92	0.0149	Rattus norvegicus similar to protein tyrosine phosphatase, non-receptor type substrate; brain immunological-like with tyrosine-based motifs (LOC310212)
Hck	3.87	0.0100	hemopoietic cell kinase
Trem2	3.78	0.0100	triggering receptor expressed on myeloid cells 2
Ccl6	3.71	0.0100	Rattus norvegicus chemokine (C-C motif) ligand 6
Cd36	3.57	0.0100	CD36 antigen
Igf1	3.23	0.0100	insulin-like growth factor 1
Ctss	3.15	0.0100	cathepsin S
Gzmc	3.11	0.0373	granzyme C
LOC100048479	2.97	0.0373	one cut domain, family member 1
Plscr1	2.83	0.0100	phospholipid scramblase 1
Nfe2	2.74	0.0149	nuclear factor, erythroid derived 2
Prg4	2.74	0.0149	proteoglycan 4
Spic	2.68	0.0278	Spi-C transcription factor
Fcgr2b	2.62	0.0100	Fc receptor, IgG, low affinity IIb
LOC498277	2.61	0.0100	similar to Low affinity immunoglobulin gamma Fc region receptor III precursor

The percentage of significantly (p<0.05) and strongly (log2-fold change ≥1 or ≤−1) up- or down-regulated genes was higher in the NKC region (14.0%) compared to MHC region (4.9%) and the genes encoded in other regions of the genome (2.6%). This difference was even more pronounced for up-regulated genes. 14.0% of the NKC, but only 4.5% of the MHC and 1.5% of the other genes were up-regulated (**Table 2**).

**Table 2:** Proportion of regulated genes as indicated by the gene expression profiling experiment.

region	analyzed genes	regulated^1^	up-regulated	down-regulated
**MHC**	224	11 (4.9%)	10 (4.5%)	1 (0.4%)
**NKC**	43	6 (14.0%)	6 (14.0%)	0 (0%)
**others**	6342	168 (2.6%)	93 (1.5%)	75 (1.2%)

1 Only those genes that were both significantly (p<0.05) and strongly (log2-fold change ≥1 or ≤−1) regulated were taken into account for this comparison.

For a general analysis of the gene expression data the PANTHER system [23] was used. With this tool we found a significant up-regulation of genes taking part in “immunity and defence” (p<0.0001, binominal test). More specifically, genes involved in “T cell-mediated immunity” (p<0.0001), “NK cell-mediated immunity” (p<0.0001), “cytokine and chemokine-mediated signaling” (p = 0.0032), and “B cell and antibody-mediated immunity” (p = 0.0235) were up-regulated. Genes involved in “complement-mediated immunity” (p = 0.0336) and “cell adhesion“ (p = 0.0003) were down-regulated (data not shown).

### Confirmation of microarray results by quantitative real-time polymerase chain reaction (qRT-PCR)

To determine the reliability of the microarray results, we analyzed the expression of 13 selected genes from the MHC and NKC regions by qRT-PCR experiments in 8 of the sample pairs that had been used for the microarrays (see **Fig. 1**). For 12 genes the regulation that was observed in the microarray experiment was confirmed by qRT-PCR as indicated by a regulation into the same direction when the allogeneic and syngeneic skin explant assays were compared using the ΔΔ cycle threshold (ct) method for relative quantification of gene expression (**Fig. 3**). Only one gene, *RT1-CE10*, was found to be strongly up-regulated in allogeneic skin explants in the microarray experiment but slightly down-regulated in qRT-PCR. In the qRT-PCR experiments, we also included parallel skin explants that were cultured in medium only. Eight genes (*RT1-DMb*, *Aif1*, *Lst1*, *RT1-CE3*, *Ubd*, *Olr1*, *Ly49si1*, and *Ly49i9*) showed an up-regulation in the allogeneic skin explant assay also in this comparison (**Fig. 3**). Six of these genes (*Aif1*, *Lst1*, *Ubd*, *Olr1*, *Ly49si1*, and *Ly49i9*) were clearly found to be up-regulated in both comparisons.

**Figure 4:**
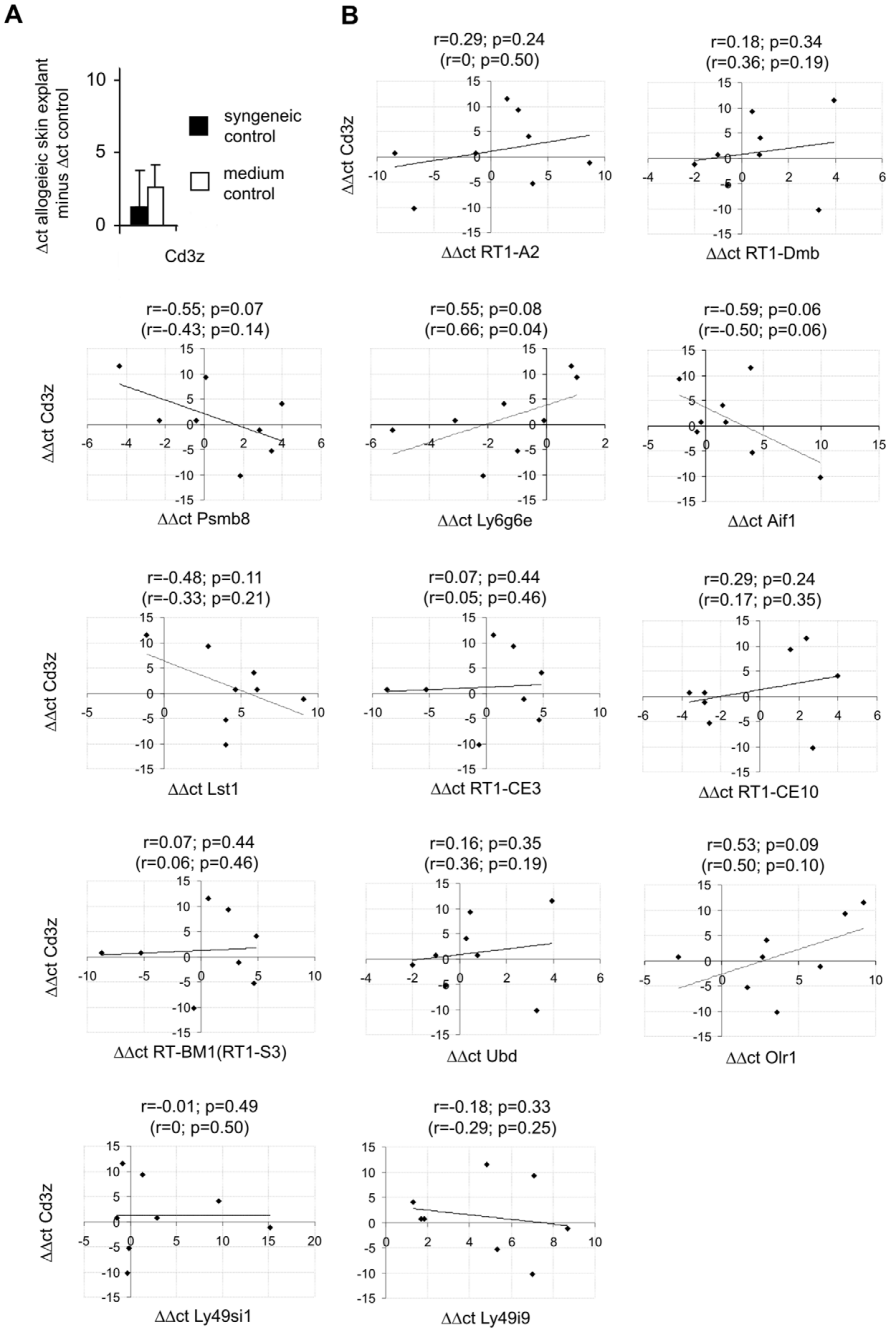
Analysis of T cell infiltration in skin explants. (***A***) Analysis of *Cd3z* gene expression in the same samples as shown in **Fig. 3**. (***B***) Correlation of *Cd3z* and other gene expression levels (ΔΔct values for allogeneic skin explants minus syngeneic controls) in these samples. Pearson's correlation coefficients (r) and the p-values for the corresponding tests are given above the diagrams. In brackets Spearman's correlation coefficients (r) and the p-values for the corresponding tests are shown.

The up-regulation of genes in skin explants could be due to the change of gene expression in cells of the skin or due to infiltration of donor lymphocytes. Non-infiltrating or non-attaching donor lymphocytes were washed off before freezing of the skin explants and therefore would not contribute significantly to the results. Infiltrating lymphocytes were rarely seen in skin explants by histological analysis (data not shown). To further determine T cell infiltration at the RNA level, we analyzed the expression of the CD3 zeta chain in qRT-PCR. *Cd3z* expression was found to be up-regulated in comparison to syngeneic controls and medium controls (**Fig. 4** ***A***). The expression of most tested genes showed no correlation with *Cd3z* mRNA levels (**Fig. 4** ***B***). Only two of the genes analyzed in qRT-PCR (*Ly6g6e* and *Olr1*) showed a moderately positive correlation (r>0.50) with the *Cd3z* expression level (**Fig. 4** ***B***). Importantly, *Ly6g6e* was down- and not up-regulated in allogeneic skin explants. The expression levels of three up-regulated genes (*Psmb8*, *Aif1*, and *Lst1*) were even negatively associated with *Cd3z* expression (**Fig. 4** ***B***). Thus, of the tested genes only the increase of *Olr1* expression may be formally explained by infiltrating T cells. However, *Olr1* has not been described to be expressed in T cells. Therefore, infiltration of skin explants with T cells is unlikely to explain the observed gene expression changes.

### Analysis of microarray results by qRT-PCR in independent skin explant samples

**Figure 5:**
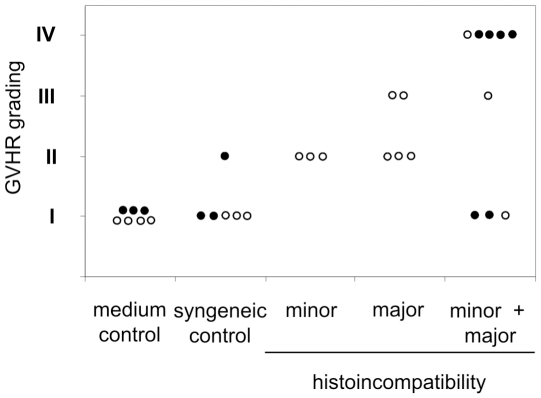
Induction of a GVHR in a second series of BN (filled circles) and LEW.1N (open circles) rat skin explants. Skin explants were co-cultured with pre-stimulated allogeneic lymphocytes from rats with a minor (BN lymphocytes and LEW.1N skin), major (LEW.1A (*RT1^a^*) or LEW.1AV1 (*RT1^av1^*) lymphocytes and LEW.1N skin), or a minor and major histoincompatibility (PVG lymphocytes (*RT1^c^*) and BN skin or LOU/C (*RT1^u^*) lymphocytes and LEW.1N skin). A summary of the histological GVHR grading of skin samples cultured in medium alone, together with syngeneic BN or LEW.1N lymphocytes, and together with allogeneic lymphocytes is given.

**Figure 6:**
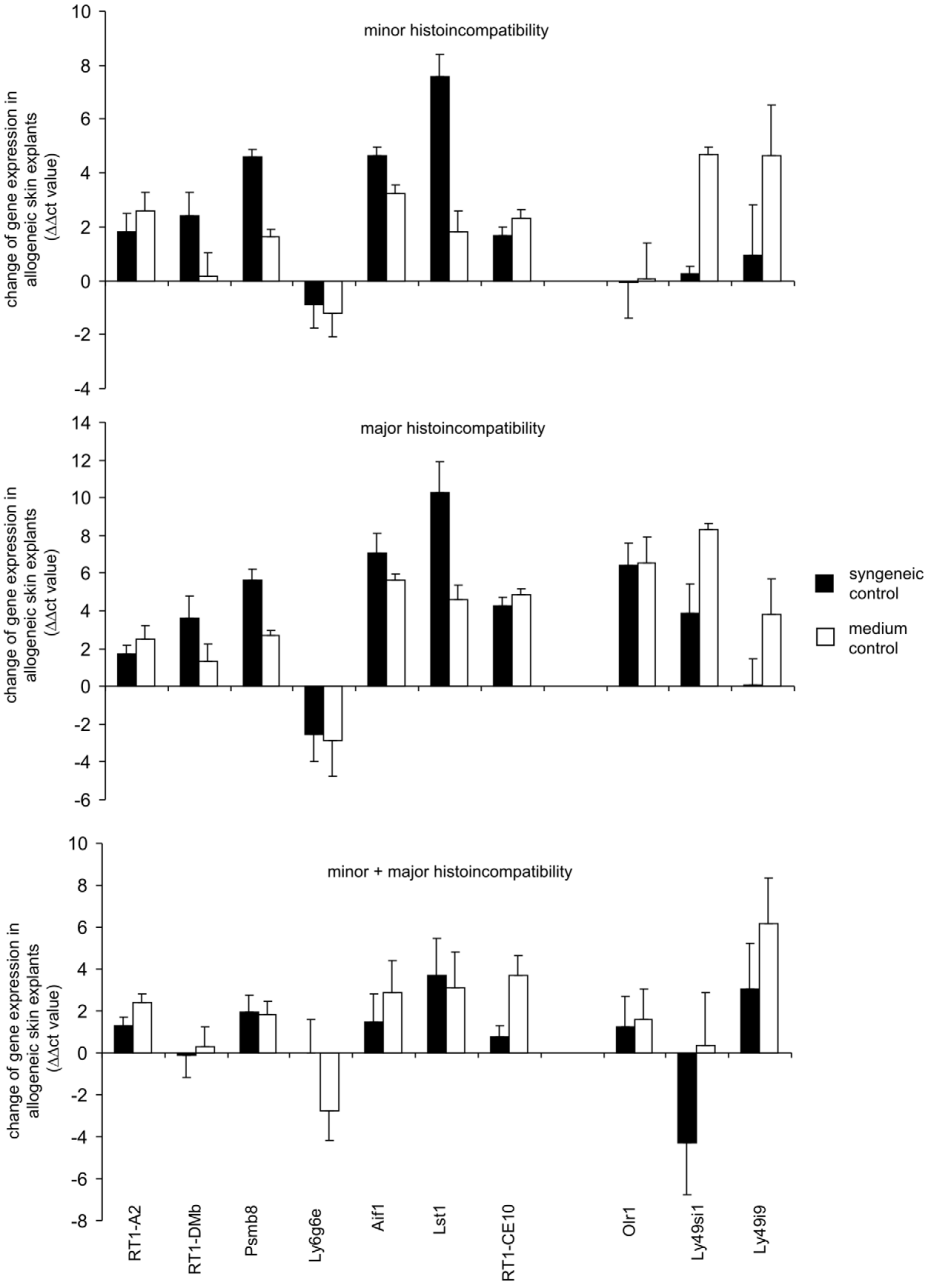
Verification of gene regulations observed in the microarray experiment by qRT-PCR in an independent set of 17 skin explant assays. Three samples were derived from skin explant assays with minor (upper panel), 5 with major (middle panel), and 9 with minor and major histoincompatibility (lower panel). The GVHR grading for these samples is shown in **Fig. 5**. The expression of 7 MHC and 3 NKC was analyzed by qRT-PCR. The ΔΔct value, i.e. Δct (*Gapdh* – gene of interest) of the allogeneic skin explant samples minus mean of Δct (*Gapdh* – gene of interest) of the corresponding control samples (BN or LEW.1N, respectively), was calculated. The control samples were either skin explant samples exposed to syngeneic lymphocytes (syngeneic control) or skin explant samples cultured in medium only without added lymphocytes (medium control) and their GVHR grading is also shown in **Fig. 5**. The means of the ΔΔct values plus SEM are shown. A positive value indicates an up-regulation of gene expression in the allogeneic samples.

Next we determined the expression of 10 selected genes in an independent set of skin explant assays. Skin explants derived from BN (*RT1^n^*) and LEW.1N (*RT1^n^*) rats were co-cultured with pre-stimulated allogeneic lymphocytes from rats with minor (BN lymphocytes and LEW.1N skin), major (LEW.1A [*RT1^a^*] or LEW.1AV1 [*RT1^av1^*] lymphocytes and LEW.1N skin), or minor and major histoincompatibility (PVG lymphocytes [*RT1^c^*] and BN skin or LOU/C [*RT1^u^*] lymphocytes and LEW.1N skin). Skin samples cultured with syngeneic lymphocytes (BN or LEW.1N) or cultured in medium only served as controls. The GVHR grading obtained in these experiments is shown in **Figure 5**. The general regulation of the selected genes during GVHR was reproduced in this second experimental set when compared to skin explants exposed to syngeneic lymphocytes and also to samples cultured in medium only (**Fig. 6**). *Aif1* and *Lst1* were the most consistently up-regulated genes in skin explants with minor, major, and minor plus major histoincompatibility. The samples with minor plus major histoincompatibility showed the highest variation in gene regulation (**Fig. 6**). However, these samples were also most heterogeneous in the GVHR grading (**Fig. 5**). Therefore, we analyzed the gene regulation dependent from the GVHR grading in samples from both experimental sets.

### Regulation of selected MHC and NKC genes during GVHR

**Figure 7:**
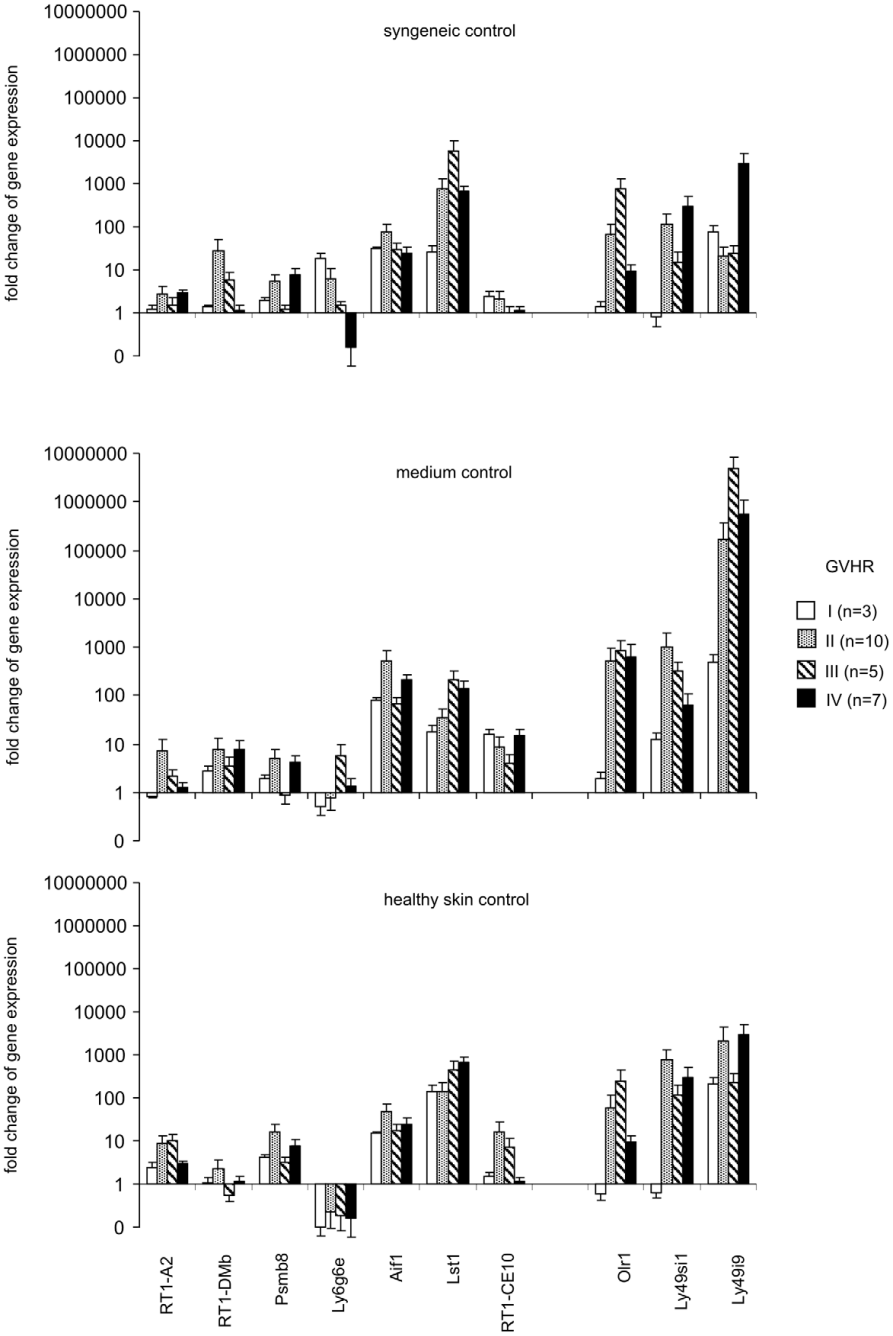
Analysis of MHC and NKC gene regulation in skin explants exposed to pre-stimulated allogeneic lymphocytes depending on GVHR grading. The expression of 7 MHC and 3 NKC was analyzed by qRT-PCR. The relative changes of gene expression levels were calculated using a mathematical model for relative quantification of real-time PCR data which also takes into account variations of the amplification efficiencies of different primer pairs [24]. The means plus SEM are shown. A value >1 indicates an up-regulation of gene expression in the allogeneic samples. The control samples were either skin explant samples exposed to syngeneic lymphocytes (syngeneic control, upper panel), skin explant samples cultured in medium only (medium control, mean panel), or freshly frozen healthy skin samples (healthy skin control, lower panel).

The expression of 7 MHC and 3 NKC genes was evaluated in the skin explant samples showing grade I, II, III or IV GVHR (**Fig. 7**). To provide an even more accurate comparison of the different genes in this evaluation of the data, the relative changes of gene expression levels were calculated using a mathematical model for relative quantification of real-time PCR data which takes into account variations in the amplification efficiencies of different primer pairs [24]. When compared to skin explants exposed to syngeneic lymphocytes or to medium controls, the genes *Aif1*, *Lst1*, *Olr1*, and *Ly49i9* were consistently up-regulated. *Ly6g6e* was down-regulated in some but not all comparisons. The expression of *Aif1*, *Lst1* and *Ly49i9* was found to be increased in all GVHR grades. The extremely high up-regulation of *Ly49i9* encoding an NK receptor in comparison to medium controls might be explained by complete absence of NK cells in normal skin biopsies and infiltration of few NK cells during GVHR. When the gene expression was compared to freshly frozen healthy skin, the principal findings were confirmed. Interestingly, *Olr1* was up-regulated mainly in grade II and III GVHR samples when compared to syngeneic control skin explants and healthy skin. Thus, this gene could be a marker of intermediate grade GVHR.

### Regulation of selected MHC and NKC genes during GVHD

**Figure 8:**
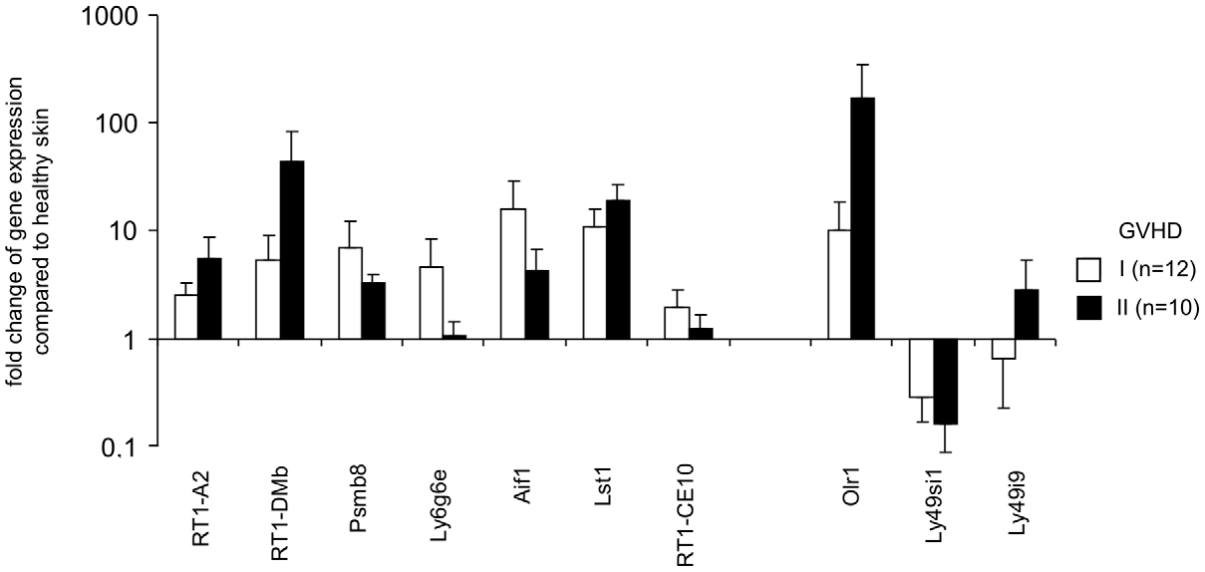
Analysis of MHC and NKC gene regulation in GVHD skin lesions from transplanted animals. BN (*RT1^n^*) rats were transplanted with bone marrow of PVG (*RT1^c^*) rats. Rats that developed acute GVHD were scarified and skin lesions with signs of GVHD were obtained for RNA preparation and histology. The expression of 7 MHC and 3 NKC was analyzed by qRT-PCR using the *B2m* gene as reference. The relative changes of gene expression levels were calculated [24]. The means plus SEM are shown for skin lesion with grade I and grade II GVHD. A value >1 indicates an up-regulation of gene expression in the allogeneic samples. The control samples were freshly frozen skin samples from healthy BN rats (n = 7).

Next, we wanted to know whether the genes found to be differentially expressed in GVHR in skin explant assays were also regulated *in vivo* in GVHD. For this purpose we analyzed skin samples from BN rats that were transplanted with bone marrow from PVG rats and developed acute GVHD. The analyzed skin samples showed in histology a grade I or grade II GVHD. The results of qRT-PCR for 7 MHC genes and 3 NKC genes are shown in **Figure 8**. The strongest up-regulation in GVHD-affected skin was observed for *RT1-DMb*, *Aif1*, *Lst1*, and *Olr1*. Thus, most genes that were found to be regulated in GVHR in skin explants were also regulated in GVHD-affected skin. However, the *Ly49si1* gene that was up-regulated consistently in allogeneic skin explants showing GVHR of grade II and above appeared to be down-regulated in GVHD. Compared to the skin explant samples, also the *Ly49i9* gene was only moderately up-regulated in grade II GVHD samples from transplanted rats.

### Regulation of selected MHC and NKC genes during GVHR in human skin explant assays

Finally, we explored the regulation of the identified genes during GVHR in human skin explant assays. We determined the expression of those genes for which human homologues exist. At 1, 2 and 3 days of co-culture with alloreactive lymphocytes skin samples of one donor were taken and analyzed in comparison to parallel samples cultured in medium only. At day 1 a GVHR of grade I was observed that increased to grade II at day 2 and grade III at day 3. We determined the expression of 15 MHC and 1 NKC gene by qRT-PCR (**Table 3**). Of these 16 genes 12 (75%) were regulated at least in one skin explant sample in the way predicted by the results of the rat expression profiling experiments (**Table 4**). Three genes *TAP1*, *PSMB8*, and *UBD* were up-regulated in all 3 human skin explant samples. The genes *C2*, *FLI13158*, and *OLR1* were regulated in 2 of the 3 samples as predicted by the rat experiments. In addition, we determined the expression of 153 non-MHC/non-NCR genes that were identified to be regulated in rat skin explant assays. Also of these genes 105 (69%) were regulated in at least one of the human skin explant samples in accordance with the results obtained in the rat model (**Table 4**). These results suggest that the rat model of the skin explant assay can reliably predict gene expression changes that occur also in human skin explant assays during GVHR.

**Table 3:** Regulation of MHC and NCR candidate genes in human skin explants.

	regulation in rat skin explant assays (expression profiling)	regulation in human skin explant assay			concordance rate
		day 1(GVHR I)	day 2(GVHR II)	day 3(GVHR III)	
**MHC region**					
HLA-DMB	↑^1^	-	↑	-	1/3
TAP1	(↑)	↑	↑	↑	3/3
PSMB8	↑	↑	↑	↑	3/3
G18 (GPSM3)	↑	n.d.	n.d.	n.d.	
PBX2	(↑)	↓	n.d.	↑	1/3
C2	↑	↑	↑	↓	2/3
LY6G6E	↓	n.d.	↑	n.d.	0/3
BAT5	↓	-	-	-	0/3
AIF1	↑	↓	↑	↓	1/3
LST1	↑	-	↑	n.d.	1/3
SPR1 (PSORS1C2)	↑	-	-	↑	1/3
IER3	↑	↓	↑	-	1/3
FLI13158	(↓)	↓	↓	-	2/3
MRPS18B	(↑)	↓	↓	↓	0/3
UBD	↑	↑	↑	↑	3/3
**NCR region**					
OLR1	↑	↑	↑	n.d.	2/3

1 Explanation of symbols:

↑ up-regulated mRNA expression level (log2-fold change ≥1).

↓ down-regulated mRNA level (log2-fold change ≤−1).

- unchanged mRNA expression level (log2-fold change >-1 and <1).

(↑) significant (p<0.05) but moderate up-regulation (log2-fold change <1) of mRNA expression level in the rat expression profiling experiment.

(↓) significant (p<0.05) but moderate down-regulation (log2-fold change >−1) of mRNA expression level.

n.d. no mRNA detected.

**Table 4:** Proportion of concordantly regulated in MHC, NKC, and genes encoded in other regions in human skin explant assays in comparison to rat skin explant assays.

region	analyzed human genes	mRNA not detected	concordantly regulated in human skin explant assays in comparison to rat skin explant assays			not concordantly regulated
			3/3	2/3	1/3	0/3
**MHC**	15	1 (7%)	3 (20%)	2 (13%)	6 (40%)	3 (20%)
**NKC**	1	0 (0%)	0 (0%)	1 (100%)	0 (0%)	0 (0%)
**others**	153	18 (12%)	33 (22%)	31 (20%)	41 (27%)	30 (20%)

## Discussion

We aimed to identify genes that are regulated during GVHR in the skin explant assay because these genes could be involved in the pathophysiology of GVHR and contribute to the genetic risk of GVHD. Special attention was given to genes encoded within the MHC region for the following reasons: Firstly, evidence has been presented that further risk genes for GVHD in addition to MHC class I and class II genes are present in this region [12]. Secondly, those genes cannot easily be identified by genetic linkage analysis alone due to the strong linkage disequilibrium with MHC class I and class II genes so that expression profiling could be a worthwhile alternative approach. Thirdly, we wanted to focus in this initial study on a fully characterized genomic region of special immunological importance rather than to follow a whole genome expression profiling approach. Importantly, 39% of the BN rat MHC genes (*RT1^n^* haplotype) annotated by Hurt and colleagues [16] were at the time point of array construction not represented in the Agilent database and therefore not represented on the Agilent whole rat genome array. In addition to the MHC region, genes of the NKC region were included because this region encodes *Ly49* genes and their products can function as receptors for the numerous MHC class Ia and Ib gene products encoded in the MHC [17].

A higher percentage of MHC genes and NKC genes than genes in other regions of the genome were found to be regulated in the allogeneic skin explants compared to skin samples co-cultured with syngeneic lymphocytes. Of the 25 MHC genes found to be significantly regulated (p<0.05), 5 are known to be involved in antigen processing and presentation. Besides two of three MHC class Ia genes in the BN strain (*RT1-A1* and *RT1-A2*) that present peptides to cytotoxic T lymphocytes (CTL), the genes *Tap1* and *Psmb8*, encoding a subunit of the antigen transporter and a subunit of the immunoproteasome (also known as LMP7), were found to be up-regulated. *RT1-DMb* encodes a homologue of *HLA-DMB*, a chaperone in the MHC class II presentation pathway. Furthermore, non-classical MHC class Ib genes (*RT1-CE2*, *RT1-CE3*, *RT1-CE5*, *RT1-CE8*, *RT1-CE10*, *RT1-CE16*, *RT1-T24-4*, *RT-BM1*) were up-regulated during GVHR in the skin explants. The function of the RT1-C/E/M class I genes is not well defined. It is known that they can become targets of CTL [25] and function as ligands for activating or inhibitory NK receptors [17], [18]. RT1-C/E/M incompatibility has been shown to induce skin and pancreas graft rejection [26] and to modulate the fate of MHC class II-mismatched heart grafts [27]. The *RT1-T24-4* gene belongs to a family of genes that was originally identified as pseudogenes in the haplotype *r21* [28]. In the *RT1^n^* haplotype all four family members are presumably functional [16]. However, their actual function has not been experimentally demonstrated so far. The *RT-BM1* (*RT1-S3*) gene is assumed to be orthologous to the mouse *H2-T23* gene [29], [30], which encodes the Qa-1 molecule. This is a functional homologue of HLA-E, which presents leader peptides of MHC class I molecules to the inhibitory NK receptor CD94/NKG2A [31]. Interestingly, its expression can vary substantially depending on the RT1 haplotype [14]. It has to be noticed that no human/rat orthology can be established for the class I genes in the various class I clusters. Therefore, with respect to class I genes, the rat cannot serve as a model for the HLA complex. However, the non-class I genes are clearly orthologous [15], [16].

In addition to *Tap1*, *Psmb8*, and *RT1-DMb*, 12 further non-class I MHC genes were found to be regulated in the rat skin explant assays, some of them also involved in the immune response, such as the complement component *C2*, while such a role is strongly assumed for other genes. The allograft inflammatory factor 1 (*Aif1*), was cloned from chronically rejecting rat cardiac allografts [32] and it was also found in transplanted human hearts [33]. Persistent expression of AIF-1 is associated with the development of a cardiac allograft vasculopathy [34]. The expression of AIF-1 is mostly limited to the monocyte/macrophage lineage, and can be augmented by interferon (IFN)-γ. The specific function of the leukocyte specific transcript 1 (*Lst1*) gene is not known, although its strong expression in dendritic cells and functional data suggest an immunomodulatory role [35]. The expression of human *LST1*, specifically of splice variants encoding soluble isoforms, was increased in rheumatoid arthritis-affected blood and synovium and was up-regulated in response to IFN-γ [36]. The immediate early response 3 (*Ier3*) gene is stress-inducible and is involved in the regulation of cell death and oncogenesis [37]. The protein (also known as IEX-1 or IEX-1L) functions in the protection of cells from Fas or TNF-α-induced apoptosis [38]. However, it increases the rate of apoptosis in ultraviolet B irradiated keratinocytes [39]. Distinct domains of the proteins were described to be responsible for pro and anti-apoptotic activities of the protein [40]. The diubiquitin gene (*Ubd*) has been shown to be expressed in rat lymphoblasts, thymus, and testis [41]. In the mouse it is expressed in dendritic cells and B cells, is inducible by IFN-γ, and can cause apoptosis [42]. The protein (also known as FAT10) provides an ubiquitin-independent signal for proteasomal degradation [43]. It has been suggested to participate in antigen processing [44], but its expression did not affect MHC class I expression or antigen presentation [42]. In view of the reported roles of these genes in the immune response, a direct involvement in GVHD is conceivable.

For the other regulated MHC genes an involvement in immune functions has not been established so far. *Spr1* (or *Psors1c2*) is the psoriasis susceptibility 1 candidate 2 gene and was found to be expressed in the thymus of rats [41]. Its human homologue is expressed in normal and psoriatic skin and has been suggested to confer susceptibility to psoriasis [45]. The function of the gene product is not known so far. *G18* (*Gpsm3*) is an activator of G-protein signaling [46]. *Pbx2* encodes an ubiquitously expressed transcriptional activator [47]. The *Ly6g6e* gene belongs to the lymphocyte antigen 6 (Ly-6) superfamily that encodes proteins attached to the cell surface by a glycosylphosphatidylinositol (GPI) anchor that is directly involved in signal transduction [48]. Mouse Ly6g6e was found to be highly expressed at the leading edges of cells, on filopodia, which are normally involved in cell adhesion and migration [49]. The mitochondrial ribosomal protein S18B (*Mrsps18b*) gene encodes a 28S subunit protein that belongs to the ribosomal protein S18P family. The functions of the HLA-B associated transcript 5 (*Bat5*) and *Fij13158* (or *RGD1303066*) genes have not been characterized so far. Some MHC genes, such as *Tnf* encoding the tumor necrosis factor alpha or the heat shock protein 70 genes *Hspa1b* and *Hspa1a*, which were expected to be up-regulated [22], [50], were actually not found to be significantly regulated in the rat skin explants. Thus, it is possible that some MHC genes that can be regulated during GVHR were not identified in our microarray experiments.

Many of the up-regulated MHC genes are inducible by IFN-γ a type II cytokine that is primarily secreted by activated T and NK cells. Several studies have demonstrated an increased level of IFN-γ in the early phase of GVHD [51], [52]. Therefore, this cytokine might be highly important for the regulation of the expression of MHC genes during GVHR.

We also included the NKC region in the expression profiling which harbors the Ly49 genes that encode NK receptors of the killer cell lectin-like receptor type [19] and some of these have been shown to interact with both MHC class Ia and Ib molecules [17], [18]. In contrast to the MHC region, no reference sequence has been published for the NKC region of the rat. Therefore, 20 genes that were recently assigned to this region in the assembly RGSC v3.4 [53] were not represented on the array. However, for most of them no function associated with the immune system has been reported. Interestingly, only *Ly49* receptor genes which have an ITIM motif in their cytoplasmic region were up-regulated in the allogeneic skin explant assays. This includes also the *LOC690045* gene which encodes an immunoreceptor similar to *Ly49si1*. It is not clear whether one of these gene products interacts with the MHC class Ib molecules that we found to be up-regulated. Ly49 receptors are normally present mainly on NK cells and the skin explants harbored few leukocytes. However, skin resident lymphocytes can become activated in human skin explant assays [54]. Although few NK cells infiltrating a tissue that normally does not contain these cells might cause a drastic relative change in the presence of *Ly49* transcripts, the possibility should not be dismissed that other cells may express the receptors under pathological conditions. The role of NK cells for GVHR in skin explants needs to be further explored. In general NK cells are assumed to prevent GVHR, improve engraftment and to exert strong graft-versus-leukemia effects without causing GVHD [55].

In the NKC region we found one non-*Ly49* gene to be regulated. The *Olr1* gene encodes a receptor protein which belongs to the C-type lectin superfamily. The protein (also known as LOX-1) binds, internalizes and degrades oxidized low-density lipoprotein, which induces vascular endothelial cell activation and dysfunction, resulting in pro-inflammatory responses, pro-oxidative conditions and apoptosis [56]. In addition, it acts as a receptor for extracellular heat shock protein 70 on dendritic cells. Binding and internalization of heat shock protein 70/peptide complexes channels peptides into the MHC class I presentation pathway [57]. Thus, the protein is involved in antigen cross-presentation to naive T cells.

In addition to the MHC and NKC region genes, 168 further genes were significantly regulated in allogeneic skin explants. Many of them also have immunological functions and need to be analyzed in more detail in subsequent studies.

The results obtained in the MHC and NKC gene expression profiling experiment were confirmed in most tested cases by qRT-PCR on the skin explant samples. Some genes, e.g. *Aif1* and *Ly49i9*, appeared to be up-regulated even in grade I GVHR. *Olr1*, in contrast, was up-regulated predominantly in grade II and III GVHR in all comparisons. Importantly, several of the MHC and NKC genes that were identified to be regulated in the skin explant assays, including *Aif1*, *Lst1*, and *Olr1*, were also regulated in the GVHD affected skin of transplanted animals. Thus, the skin explant assay can model GVHD not only histologically but also with respect to gene regulation. However, the up-regulation of the tested *Ly49* genes (*Ly49si1* and *Ly49i9*) that were observed in the skin explant was not clearly confirmed in the GVHD-affected skin of transplanted rats. Skin lesions from transplanted animals are likely to be more heterogeneous with respect to the dynamics of the pathophysiological process, including infiltration of NK cells, than skin explant samples, and this may contribute to the variation in results. In addition, variability between microarray and qRT-PCR results might be partly attributed to the fact that in qRT-PCR experiments the results were normalized to only one control gene. In contrast, many genes were used in the microarray experiments for data normalization. More control genes could be employed for data normalization in qRT-PCR experiments in further validation studies to reduce this source of variability [58].

In an exploratory experiment, we analyzed the expression of 169 genes with human homologues, including the respective MHC and NKC region genes, identified in the rat in human skin explant samples. These human skin explants were cultured for 1, 2, or 3 days resulting in GVHR of grades I, II, and III, respectively. Notably, 69% of all tested human genes were found to be regulated in at least one of these human samples as predicted by the results of the rat expression profiling experiments. 21%, i. e. 36 of the tested genes, were regulated in all 3 human skin explant samples in accordance with the rat model, but this regulation varied depending on the GVHR grade and the time course of the skin explant assay. Although we only validated these genes on 3 samples, the unexpectedly high concordance rate between the results of rat and human skin explant assays strongly suggests that the rat skin explant assay is an informative model for human GVHR and possibly GVHD. However, the variability of results in these few human skin explant samples also indicates that it is unlikely to find a single gene that can serve as universal marker for GVHR. It is more conceivable that patterns of gene expression could contribute to an improved diagnosis and classification of GVHR.

Interestingly, for some of the genes that we found to be regulated in GVHR and GVHD in the rat, the human homologues are polymorphic and disease associations of gene polymorphisms have been described. These include *HLA-DMB* [59], *C2* [60], *AIF1* [61], *SPR1* [45], and possibly *UBD* [62]. Therefore, these genes are especially interesting candidates of further non-class I/class II HLA genes that might confer an increased genetic risk of GVHD after HSCT depending on the genotype. In addition, the *OLR1* gene in the NKC is polymorphic and polymorphisms of this gene have been associated with atherosclerosis, myocardial infarction [63], and Alzheimer's disease [64].

Several laboratory tests have been assessed for their ability to predict the risk of GVHD in patients. The skin explant assay has a predictive value of about 80% when cyclosporine alone is used for GVHD prophylaxis [65]. A gene expression analysis of selected genes may help to further improve the predictive value of the assay. Pre-transplant gene expression profiling of donor peripheral blood mononuclear cells (PBMC) has recently been shown to be a useful tool to predict the risk of GVHD [66]. Post transplant differences in the gene expression profile of PBMC of patients with acute [67], [68] and chronic GVHD [69] compared to non-GVHD samples have been described. The general gene expression profile of target tissues of GVHD has been previously analyzed only in mouse models of cutaneous and hepatic GVHD [70], [71]. Some of the genes identified in these studies overlap with our results. In cutaneous GVHD the MHC genes *Tap1*, *Psmb8*, and *Ubd* were also found to be up-regulated [71]. In hepatic GVHD the expression of *Tap1*, *Psmb8* (*Lmp7*), *H2-DMb*, *Aif1*, and *Ubd* (*Fat10*) was increased [70].

In conclusion, the MHC gene expression profiling approach in the rat skin explant assay identified a number of non-class I/class II genes that might contribute to the MHC-associated risk of GVHD following HSCT. These genes could be directly involved in the pathophysiology of GVHD or serve as molecular markers for GVHD and GVHR. The possibility should not be dismissed, however, that these marker genes could indicate that protective pathways are induced which modulate tissue damage during inflammation. Moreover, their human homologues may be useful for risk assessment, diagnosis, and as potential targets for therapy of GVHD in patients.

## Materials and Methods

### Rat strains

For the skin explant assays, rats of the inbred strains LEW.1N (*RT1^n^*), LEW.1A (*RT1^a^*), LEW.1AV1 (*RT1^av1^*), LOU/C (*RT1^u^*), and BUF (*RT1^b^*) were bred in the central animal facility of the Medical Faculty of the University of Göttingen. Rats of the strains PVG/OlaHsd (*RT1^c^*) and BN/RijHsd (*RT1^n^*) were purchased from Harlan Winkelmann (Borchen, Germany). Animals between 10 and 20 weeks of age were used for the experiments. For transplantation experiments, PVG rats of the RT7.2 allotype (allelic variant *RT7^b^*), originally obtained from Harlan OLAC, UK), were bred at the animal facility of the University of Oslo and BN rats were purchased from Harlan.

### Rat skin explant assays

Rat skin explant assays were performed as previously described in detail [22]. Briefly, mononuclear cells were obtained from rat spleens. Responder and irradiated (25 Gy) stimulator splenocytes were co-cultured in a MLR and the proliferation of responder lymphocytes was tested by [methyl-^3^H]-thymidine (Amersham, Braunschweig, Germany) incorporation. The stimulation index was calculated as described [22]. After 7 days 10^6^ responder lymphocytes were added to freshly obtained skin samples from the stimulator strain that were cultured in 200 µl NaHCO_3_-buffered Dulbecco's modified Eagle's medium (DMEM; Biochrom) supplemented with 3% normal rat serum, 2 mM L-glutamine, 1 mM sodium pyruvate, and antibiotics in round-bottomed microtitre plates (Sarstedt, Nümbrecht, Germany). The skin samples were excised from the paws of rats after washing with 70% ethanol. The subcutaneous fat tissue was removed and the samples were trimmed to a size of approximately 1.5×1.5 mm. Skin samples cultured in medium only and samples co-cultured with lymphocytes from a “syngeneic MLR” were used as controls. After 3 days, the skin explants were washed with *N*-2-hydroxyethylpiperazine-*N*'-2-ethanesulfonic acid (HEPES)-buffered DMEM and snap frozen in liquid nitrogen and stored at −80°C for RNA preparation. Parallel samples were fixed in 10% neutral-buffered formalin, sectioned, and stained with hematoxylin and eosin (H&E). The histological evaluation of the skin explants was performed blind by an expert histopathologist (L.S.) based on the grading system described by Lerner [72].

### Human skin explant assays

PBMC and skin samples were obtained from healthy volunteers or autologous HSCT patients following written informed consent and approval from the North Tyneside Research Ethics Committee. Buffy coat from normal blood donations were obtained from Newcastle National Blood Service with consent. Skin explant assays were performed as previously described with slight variations [73]. In brief, 1×10^7^ responder PBMC from a healthy volunteer were cultured with an equal number of irradiated PBMC from a bone marrow transplant patient, in 10 ml complete medium (RPMI 1640 supplemented with antibiotics, 2 mM L-glutamine and 10% heat inactivated human AB serum) in a 25 cm^2^ flask. After 7 days of culture, MLR primed lymphocytes were washed and resuspended in complete medium supplemented with 20% heat inactivated autologous (patient) serum and co-cultured with patient skin explants at a cell concentration of 1×10^6^ cells/well in a volume of 200 µl/well. Standard 4-mm punch skin biopsy specimens were obtained pre-transplant from the patients. Under sterile conditions the skin biopsies were trimmed of excess dermis and divided into 8 to 10 sections of equal size. Each section was cultured separately with either MLR-primed responder cells or culture medium in 96-well round-bottomed microtitre plates. After 1, 2 or 3 days of co-culture skin explants were fixed in 10% buffered formalin, sectioned and stained with H&E. The histopathological evaluation [72] of the skin explants was performed blindly and independently by at least 2 assessors. Grade I histopathological damage in skin biopsies was regarded as background which would be observed in medium control or autologous cell/autologous MLR controls. All biopsies presenting histopathological damage of grade II or above were regarded as positive.

### Bone marrow transplantation

Transplantation experiments were approved by the Experimental Animal Board under the Ministry of Agriculture of Norway (ID 09.1514, 09.1515 and VIT 09.1512). Male PVG (*RT7^b^*) rats served as bone marrow and lymph node donors. Mononuclear bone marrow cells were purified by density gradient centrifugation in Nycoprep 1.077A (Medinor ASA, Norway). The cells were depleted of T cells by magnetic separation using anti-CD5 (Ox19) and anti-αβ T cell receptor (R73) antibodies conjugated to pan-mouse IgG coated Dynabeads (Dynal Biotech ASA, Norway). This procedure reduced the CD3^+^ T cell content in the bone marrow from 3% to less than 0.3%. Male BN rats were used as recipients. They were irradiated (9 Gy) and subsequently received an i.v. injection of 30×10^6^ PVG.7b T cell-depleted bone marrow cells. 14 days post transplantation, 1.5×10^6^ lymph node cells were injected i.v. to evoke GVHD. The rats were regularly monitored for GVHD symptoms. Rats suffering from irreversible GVHD were sacrificed and skin samples were processed for RNA preparation and histology in parallel.

### RNA preparation

RNA extraction was carried out using TRIZOL reagent (Invitrogen, Carlsbad, CA, USA) according to the manufacturer's recommendations. Afterwards, the RNA samples were treated with RQ1 RNase free DNase (Promega, Madison, WI, USA) for 20 min at 37°C in order to remove genomic DNA contaminations. The RNA was then purified as described previously [22]. Quantity and quality of extracted RNA were controlled by capillary electrophoresis using the Bioanalyzer 2100 (Agilent Technologies, Santa Clara, CA, USA).

### Microarray experiment

For the expression profiling, a custom-designed oligo DNA microarray (Agilent) was used. The 15K microarray covered 224 MHC genes by 649 oligonucleotide probes and 43 NKC genes by 101 probes. These probes were spotted in triplicates. Further probes representing 6342 genes were added mainly to allow for data normalization. A two-color 12×2 paired swap design [74] using 24 arrays was applied, comparing RNA samples from 12 independent allogeneic and 12 independent syngeneic skin explant assays. Aliquots of total RNA (200 ng) were used as starting material. The “Low RNA Input linear Amplification Kit Plus, two color” (Agilent, 5188-5340) and the “RNA Spike-In Kit” (Agilent, 5188-5279) were used for cDNA synthesis and *in-vitro* transcription according to the manufacturer's recommendations. Quantity and dye incorporation rates of the amplified cRNAs were determined using the NanoDrop ND-1000 UV-VIS Spectrophotometer version 3.2.1 (NanoDrop Technologies, Wilmington, DE, USA). Afterwards, 300 ng aliquots of Cy3 and Cy5-labeled cRNAs from syngeneic and allogeneic skin explant assays, respectively, were mixed and hybridized to the microarrays. The hybridization was performed for 17 hours at 10 rpm and 65°C. After washing, Cy3 and Cy5 intensities were detected by two-color scanning using a DNA microarray scanner (Agilent, G2505B) at 5 micron resolution. Scanned image files were visually inspected for artifacts. The generated raw data were extracted using the Feature Extraction 9.1 software (Agilent). The normalization of the raw microarray data was done with a non-linear loess regression [75]. Differentially expressed genes were identified by an analysis of variance (ANOVA) mixed effects model [74] using SAS PROC MIXED. The resulting p-values were adjusted with the Benjamini-Hochberg method to control the false discovery rate [76]. The microarray data were generated conforming to the MIAME guidelines and have been deposited in NCBI's Gene Expression Omnibus (accessible through GEO series accession number GSE17928). For a general analysis of the gene expression data the PANTHER (Protein ANalysis THrough Evolutionary Relationships) system [23] was used, which classifies genes by their functions (www.pantherdb.org/tools/genexAnalysis.jsp). The microarray data were mapped to PANTHER molecular function and biological process categories, as well as to biological pathways [77].

### Validation of rat candidate genes by quantitative real-time PCR

To validate the expression change of candidate genes, qRT-PCR assays were used. Specific primers for 10 MHC and 3 NKC genes were designed (**Table S4**). To generate external standard curves and to calculate the amplification efficiency of each primer pair, a pool of 20 random cDNAs was amplified in serial 10-fold dilutions [24]. The amplification reactions were carried out as described previously [22] using an ABI 7500 Real-Time PCR System. The data were analyzed with the ABI 7500 SDS software (Applied Biosystems). As internal control, mRNA expression of housekeeping genes *Gapdh* (Rn_Gapd_1_SG QuantiTect Primer Assay QT00199633, Qiagen, Hilden, Germany) or *B2m* (**Table S4**) were monitored. To normalize variations in the RNA concentration in different samples, the ct values obtained in real-time PCR for the genes were corrected by the ct-value obtained for the housekeeping gene in the same sample (Δct  =  ct housekeeping gene - ct gene of interest). For direct comparison with microarray data, the relative changes of mRNA expression were calculated using the ΔΔct method (ΔΔct  =  Δct sample of interest – Δct control sample) [78]. For additional analyses, the relative changes of gene expression levels were calculated using a mathematical model for relative quantification of real-time PCR data which takes into account variations of the amplification efficiencies of different primer pairs [24].

### Validation candidate genes in human skin explant assays by quantitative real-time PCR

Validation of the rat candidate genes with human homologues in the human skin explant assay was also done by qRT-PCR. For this we used relative quantification using custom designed Taqman low density array (TLDA) cards (Applied Biosystems), each card contained 4 replicates of 95 unique genes and a control gene, *18S*. The qRT-PCR reactions were set up using Taqman x2 gene expression mastermix (Applied Biosystems), 50 ng RNA equivalent of cDNA and the total volume adjusted to 200 µl with nuclease free water (Quiagen). The TLDA cards were run on a 7900 qRT-PCR system (Applied Biosystems) using the TLDA block and analysed using the RQ manager 1.2 software (Applied Biosystems). The relative changes in RNA expression were also calculated using the ΔΔct method described above.

### Statistical analyses not related to microarray experiments

Paired comparisons between experimental groups were performed using the nonparametric Mann-Whitney U test. Pearson's and Spearman's correlation coefficients were calculated to determine the correlation between mRNA expression levels of two genes. The statistical analyses were performed using WinSTAT® software.[79]

## Supporting Information

Table S1 **Expression profiling results of MHC genes.** In **Table S1a**, results for all 224 MHC genes are shown in their chromosomal order [16]. The expression profiling results of BN skin explant samples exposed to pre-stimulated allogeneic (PVG) lymphocytes in comparison to those exposed to syngeneic (BN) lymphocytes are given. The log2-fold changes and the fold changes in gene expression are shown for every oligonucleotide probe used. The adjusted p-values are indicated. Significant (p<0.05) and strong (log2-fold change ≥1 or ≤−1; i.e. fold change ≥2 or ≤0.5) results are indicated in bold font. In addition, the identification numbers of the probes on the arrays are given (probe ID) together with the information whether these probes were taken from the Agilent database or custom designed. **Table S1b** contains the same information for all MHC genes for which at least one probe indicated a significant alteration of gene expression. In **Table S1c**, the data for those genes are summarized that are considered to be regulated significantly because either at least a single probe indicated a significant (p<0.05) and strong (log2-fold change ≥1 or ≤−1) regulation or at least 50% of the gene probes indicated a significant (p<0.05) regulation of gene expression.
(XLS)

Table S2 **Expression profiling results of NKC genes.** In **Table S2a**, results for all 43 NKC genes investigated are indicated in their chromosomal order (*Klrg; Pzp* to *Csda*). The expression profiling results of BN skin explant samples exposed to pre-stimulated allogeneic (PVG) lymphocytes in comparison to those exposed to syngeneic (BN) lymphocytes are given. The log2-fold changes and the fold changes in gene expression are shown for every oligonucleotide probe used. The adjusted p-values are indicated. Significant (p<0.05) and strong (log2-fold change ≥1 or ≤−1; i.e. fold change ≥2 or ≤0.5) results are indicated in bold font. In addition, the identification numbers of the probes on the arrays are given (probe ID) together with the information whether these probes were taken from the Agilent database or custom designed. **Table S2b** contains the information for all NKC genes for which at least one probe indicted a significant alteration of gene expression. In **Table S2c**, the data for those genes are summarized that are considered to be regulated significantly because either at least a single probe indicated a significant (p<0.05) and strong (log2-fold change ≥1 or ≤−1) regulation or at least 50% of the probes indicated a significant (p<0.05) regulation of gene expression.
(XLS)

Table S3 **Regulated non-MHC non-NKC genes.** The expression profiling results of non-MHC non-NKC genes are given for those genes that were both significantly (p<0.05) and strongly (log2-fold change ≥1 or ≤−1; i.e. fold change ≥2 or ≤0.5) regulated. The log2-fold changes and the fold changes in gene expression are shown. The adjusted p-values are indicated. For 20 of these genes at least two different probes were present on the array. In 8 cases (indicated by gene symbols in bold) the second probe indicated the same strong and significant regulation and in 7 further cases (indicated by gene symbols in italics) the second probe indicated a regulation with borderline amplitude or significance. In 5 cases (indicated by gene symbols in blue font) the results of the two probes for a gene did not confirm each other. Furthermore, the identification numbers of the probes on the arrays are given (probe ID) together with the information whether these probes were taken from the Agilent database or custom designed.
(XLS)

Table S4 **Primer sequences used for mRNA expression analysis.**
(DOC)
