# Supplementary material for: Expression Profiling of Major Histocompatibility and Natural Killer Complex Genes Reveals Candidates for Controlling Risk of Graft versus Host Disease
Source: PLoS One. 2011 Jan 28;6(1):e16582. doi: 10.1371/journal.pone.0016582 (PMC3030590; doi:10.1371/journal.pone.0016582)
Supplement: Table S4 — Primer sequences used for mRNA expression analysis. (DOC) [file pone.0016582.s004.doc]

**Table S4. Primer sequences used for mRNA expression analysis**

|  | Primer sequence 5`- 3`^1^ | Amplicon  (bp) | Proximity  to poly-A (bp) | Efficiency  coefficient (*E*)^2^ |
| --- | --- | --- | --- | --- |
| *RT1-A2* | F: TCCCTCCCTGCTACCCTGAG  R: GCCATCCACACTTGGGTCAA | 103 | 105 | 1.93 |
| *RT1-DMb* | F: tcaaatctgcctcgggtgttt  R: gacaaggtggggctttcagg | 80 | 53 | 1.87 |
| *Psmb8* | F: CACTGCTGGGCAGACATCCT  R: GCTTTGTCTCCAGCCCAGGT | 109 | 91 | 1.92 |
| *Ly6g6e* | F: CCCAGGCAAAGGGACAGAAG  R: TGAGACCCTCAGGCACCAAG | 87 | 151 | 1.97 |
| *Aif1* | F: TCCCCCAGCCAAGAAAGCTA  R: TCTTTTCCCATGCTGCTGTCA | 99 | 51 | 1.86 |
| *Lst1* | F: GGGCAGGAGCTCCACTACG  R: CGATGCAGGCATAGTCAGTGC | 118 | 20 | 1.89 |
| *RT1-CE3* | F: TGTCGTCCTTGGAGCCATCT  R : TCCTCACAACAGGCACCAGA | 62 | 106 | 1.91 |
| *RT1-CE10* | F: ACACAGGTGGGGAAGGAGGA  R : CAATCTGGGAGGGACACATCAG | 82 | 10 | 1.94 |
| *RT-BM1*  *(RT1-S3)* | F: GCAGCTATGCTCATGTTCTAGGC  R: TGCCTTCTGAGGCCAGTCAG | 62 | 7 | 1.89 |
| *Ubd* | F: TGGGGTGATGAGAAGCTCAAAA  R: CCCCACCTCAAATCTTTATTTCATTC | 105 | 7 | 1.92 |
| *Olr1* | F: GGAAGTCAGAAGAGGGCATGG  R: TCCTGGGTTCAATTTCCAGAGT | 89 | 271 | 1.90 |
| *Ly49si1* | F: TGGCCAATCTGAATTTTCCTTG  R : ACATGGGAAGGGGTTCATGC | 115 | 36 | 1.84 |
| *Ly49i9* | F: GGGACTTGGCAACCTCAGGA  R: TTGGAACATCTGCACAATGGAA | 110 | 179 | 1.88 |
| *Cd3z* | F: AGTGCCTGCTGGGATTTAGC  R: CATCCATGGTCACAGGCACTT | 118 | 50 | 1.93 |
| *B2m* | F: GAGCAGGTTGCTCCACAGGT  R: CAAGCTTTGAGTGCAAGAGATTGA | 128 | 246 | 1.94 |

^1^ F: forward primer, R: reverse primer

^2^ The real-time PCR efficiency coefficient (*E*) of one cycle in the exponential phase was calculated according to the equation: *E* = 10^[–1/slope of standard curve]^
